# Cytotoxicity, antibacterial and physicochemical properties of a new epoxy resin-based endodontic sealer containing calcium hydroxide

**DOI:** 10.4317/jced.56534

**Published:** 2020-06-01

**Authors:** Emmanuel J. Silva, Fernanda Hecksher, Victor T. Vieira, Rodrigo R. Vivan, Marco A. Duarte, Sabrina C. Brasil, Henrique S. Antunes

**Affiliations:** 1 Department of Endodontics, Grande Rio University (UNIGRANRIO), Duque de Caxias, RJ, Brazil; 2 Department of Endodontics, São Paulo University, Bauru, SP, Brazil

## Abstract

**Background:**

This study evaluated the cytotoxicity, antibacterial and physicochemical properties of a new epoxy resin-based endodontic sealer containing calcium-hydroxide Sealer Plus. AH Plus was used as a reference for comparison.

**Material and Methods:**

Cytotoxicity evaluation was performed according to ISO-10993-5 specifications using MTT assay to check the 3T3 cells viability at 1- to 4-week periods. Antibacterial activity was evaluated using a direct contact test against Enterococcus faecalis. Radiopacity, solubility and flow evaluations were performed according to ISO-6876/2012 specifications. Setting time was assessed following the ANSI/ADA-standard-n.57. The pH level was measured at 3,24,48,72, and 168 hours. Data were statistically analyzed using t-test. The signiﬁcance level adopted was *P*<0.05.

**Results:**

AH Plus and Sealer Plus showed cytotoxic effects after 24 hours and 1 week of manipulation and become noncytotoxic after 2 weeks. No differences were observed in the cytotoxicity of both tested sealers (*P*>0.05). Direct contact results revealed that both freshly prepared sealers has antibacterial effects against Enterococcus faecalis. However, after 7 days both sealers had lost much of their antibacterial effects. Although AH Plus presented higher radiopacity and flow than Sealer Plus (*P*<0.05), both sealers showed minimum required values. No differences were observed in the solubility of both sealers (*P*>0.05). AH Plus showed a higher setting time when compared to Sealer Plus (*P*<0.05). AH Plus and Sealer Plus showed a neutral pH during all tested periods (*P*<0.05).

**Conclusions:**

It can be concluded that Sealer Plus showed suitable properties to be used as an endodontic sealer, comparable with those obtained by AH Plus.

** Key words:**Cytotoxicity, root canal filling materials, root canal obturation.

## Introduction

A complete sealing of the root canal system after cleaning and shaping is critical for successful endodontic therapy (1). In endodontic treatment, most root canals are filled with gutta-percha points in combination with a root canal sealer. Many different root canal sealers are currently being used in combination with gutta-percha to fill the root canal after biomechanical preparation. The characteristics and physicochemical properties of endodontic sealers are fundamental to allow hermetic sealing, which with an adequate coronal restoration will avoid bacterial leakage (2,3). Moreover, endodontic sealers are often placed in intimate contact with the periapical tissues for an extended period of time (4,5). Therefore, the biological properties, such as cytotoxicity and biocompatibility, of root canal sealers is an important factor in choosing the best material.

AH Plus (Dentsply, DeTrey GmbH, Konstanz, Germany) is an epoxy resin based endodontic sealer, considered the gold standard of endodontic sealers mainly because of its excellent physicochemical and biological properties (6,7). Recently, a new epoxy resin-based endodontic sealer was introduced on endodontic market: Sealer Plus (MKLife, Porto Alegre, RS, Brazil). According to the manufacturer Sealer Plus has excellent radiopacity, excellent flow, low solubility and low cytotoxicity. Moreover, the manufacturer claims that it has easy and quick handling, since the material (a paste-to-paste sealer) is contained in a double-body syringe which dispenses at the same time the necessary portions for the mixing of the material. This sealer has a composition similar to AH Plus, containing radiopaque fillers, calcium tungstate and zirconium oxide. The biggest difference between the sealers is the presence of calcium hydroxide in the base and catalyst past of the new sealer. Previous studies observed that the addition of calcium hydroxide to AH Plus significantly decreased the inflammation in rat subcutaneous tissue (8) without altering the sealer’s physical properties (9). It has been recently demonstrated that Sealer Plus promoted greater cell viability and was more biocompatible when compared to AH Plus, Endofill and SimpliSeal (10). However, there is a lack of information regarding other properties of Sealer Plus. Therefore, the aim of the present study was to evaluate the long-term cytotoxicity, antibacterial effects, radiopacity, solubility, flow, setting time and pH of Sealer Plus. AH Plus was used as a reference for comparison. The null hypothesis tested was that there are no differences in the tested properties between the two sealers.

## Material and Methods

Cytotoxicity evaluation was performed according to ISO 10993-5 specifications (11). Radiopacity, solubility and flow evaluations were performed according to ISO 6876/2012 specifications (12). Setting time was assessed following the ANSI/ADA standard n. 57 (13). The pH level was measured at 3, 24, 48, 72, and 168 hours. AH Plus and Sealer Plus sealers were mixed according to the manufacturer’s instructions for all the tests. The composition of the evaluated sealers are shown in Table 1.

**Table 1:**
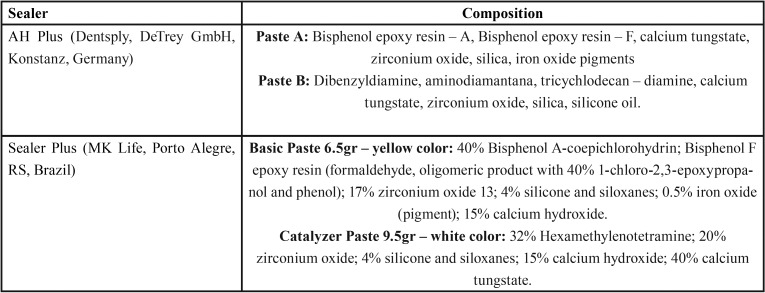
Tested sealers and their composition.1

-Cytotoxicity 

Discs of each sealer were fabricated under aseptic conditions in sterile cylindric Teflon blocks with a 5-mm diameter and a 2-mm height. Excess flash material was removed with a sterile scalpel. Cytotoxicity of the sealers was assessed after 24 hours of sealers mixing and during 4 succeeding weeks. The extraction was made in cell culture medium using a surface area to volume ratio of approximately 150 mm2/mL between the surface of the samples and the volume of the medium (11). The extraction vials were agitated for 24 hours in a water bath at 37°C. Control samples containing only culture medium were treated similarly. Undiluted extracts were used for the testing.

Fibroblast Balb/c 3T3 cells (American Tissue Type Collection, Manassas, VA) were cultured in Dulbecco modified Eagle medium (Gibco, Grand Island, NY) supplemented with 10% fetal bovine serum (Sigma-Aldrich, St Louis, MO), 100 mg/mL streptomycin, and 100 mg/mL penicillin at 37°C in a humidified incubator under ambient pressure air atmosphere containing 5% CO2. Confluent cells were detached with 0.25% trypsin and 0.05% EDTA for 5 minutes, and aliquots of separated cells were subcultured. Cells were seeded in 24-well plates (1x105 cells/well). After overnight attachment, cells were treated with the extracts of sealers (500 mL/well) for a total of 4 weeks.

Cell viability was determined using the 3-(4,5-dimethylthiazol-2-yl)-2,5-diphenyl tetrazolium bromide (MTT) assay. After the removal of culture medium from each well, the cells were gently washed with 1.0 mL phosphate-buffered saline. The wash was replaced with an MTT-succinate solution (1 mg/mL; Sigma-Aldrich, St Louis, MO) for 4 hours. After aspiration of the solution, the cell monolayers were rinsed with double-distilled water, and then the water was completely removed. Formazan crystals produced within the cells using a succinate dehydrogenase reduction of MTT were dissolved using distaining solution (isopropanol–10%NP40-0.4N HCl). Aliquots (100 µL) of the solution were then transferred from each well to a 96-well plate, and the absorbance was measured at 490 nm using a microplate reader (Urit 660; Urit, Guillin Guanxi, China). The formazan content of each well was computed as a percentage of the control group (untreated cells). All assays were repeated 3 times to ensure reproducibility.

-Direct contact test 

The direct contact test was performed as previously described (14). A 96-well microtiter plate (Sarstedt Inc, Newton, NC) was held vertically and an area of fixed size on the side wall of the wells was coated with approximately 30 µL of each endodontic sealer using an insulin syringe and a cavity liner applicator. Sealers were either tested 20 min following mixing (fresh samples) or allowed to set for 7 days at 37°C in 100% relative humidity (aged samples). Aliquots of 10 μL of Enterococcus faecalis (ATCC 29212) bacterial suspension (containing approximately 1.5 X 106 bacteria) were placed on the surface of each tested sealer. Bacterial suspensions applied to the wall of uncoated wells served as positive controls, whilst walls coated with sealers but with no bacterial suspesions were used as negative controls.

Plates were incubated at 37°C in 100% relative humidity for 60 min and then horizontally positioned so as to permit 200 µL of TSB to be added to each well. The bacterial suspension from each well was gently mixed with a pipette for 1 min and then transferred to sterile microtitre plates and subjected to 10-fold serial dillutions in sterile saline. Bacterial survival was determined by culturing 10- µL aliquots of each dilution onto Mitis Salivarius agar plates. Colony forming units were counted after incubation for 24 h at 37°C. Samples were evaluated in triplicate.

-Radiopacity Test

Cylindric samples from each material were manufactured by pouring the manipulated sealers into metallic rings measuring 10 mm in diameter and 1 mm in thickness. Five samples of each material were prepared. The filled rings were kept at 37°C for 24 hours. until the sealers were completely set. The specimens were then removed, and the thickness was checked with a digital caliper (700–126; Mitutoyo MTI Corp, Tokyo, Japan). All sealers were placed on 5 occlusal films (Insight; Kodak Company, Rochester, NY) along with an aluminum step wedge graduated from 1 to 10 mm Al (in 1-mm increments). Radiographs were taken by using a radiographic unit (XR 6010; Gnatus, Ribeirão Preto, SP, Brazil) operating at 60 kV and 10 mA, with the exposure set at 0.3 seconds and a focus-film distance of 30 cm. After processing, the radiographs were digitized by using Canon EOS XSi with the Canon 100-mm macro lens (Canon, Tokyo, Japan) and imported into Image J 1.48v program (National Institutes of Health, USA). The radiopacity value was determined according to the radiographic density, which was also converted into millimeters of aluminum. Conversion was performed as described previously (6,7).

-Flow Analysis

A final volume of 0.05 mL cement was prepared and put on a glass plate using a tuberculin syringe of 1.0 mL. At 180 ± 5 seconds after the onset of mixing, the second glass plate (50 x 50 x 3.2 mm and 20-g weight) was carefully and centrally placed on top of the sealer followed by weighting of approximately 100 g to make a total mass on the plate of 120 g. Ten minutes after the onset of mixing, the weight was removed, and the maximum and minimum diameters of the compressed sealer disks were measured with a digital caliper. Two conditions were necessary to validate the tests: the difference between the maximum and minimum diameters should not exceed 1.0 mm, and the compressed disk should have a uniform shape. If these conditions were not met, the test was repeated. Five samples for each sealer were used, and the mean of 3 measurements for each sample, expressed to the nearest millimeter, was taken as the sample flow. According to ISO 6876/2012 specifications (12) for the flow test, a disk with at least a 20-mm diameter should be obtained.

-Solubility

**Figure 1:**
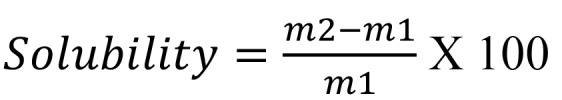
Formula.

After manipulation, sealers were placed in teflon rings with an internal diameter of 20 mm and a thickeness of 1.5 mm (n=5). A nylon thread was inserted into the material before setting, allowing the sample to be hung and immersed in distilled water throughout the experimental period. The samples were kept on a cellophane-lined glass plate, and another cellophane-wrapped glass plate was placed on the top of the filled rings. The assembly was placed in a chamber with 95% relative humidity at 37°C for 24 hours. After setting, the specimens were removed from the rings and the residues and lose particles were removed. Samples were weighed in an analytical balance with 0.001 g precision (dry mass, m1) and then placed in closed flasks with 50 mL of distilled water. Care was taken to avoid any contact between the samples and the inner surface of the container and the liquid. The specimens were then placed in a desiccator at 37°C for 48 h and reweighed again (m2). SL was calculated as: (Fig. 1).

-Setting Time Test

After manipulation, sealers were placed in stainless steel rings with an internal diameter of 10 mm and a thickeness of 2 mm (n=6). The test was performed under controlled temperature and humidity conditions: 37ºC ± 1ºC and 95% ± 5%, respectively. After 120 seconds from the start of mixing, a Gilmore-type needle with a mass of 113.5g was carefully lowered onto the surface of the samples. This was repeated until no indentation was noted on the surface of the sample. Time was recorded from the start of mixing to this point to the nearest minute.

-pH Analysis

Shortly after manipulation, the sealers were carefully placed in plastic tubes (polyethylene) measuring 1.0 mm in internal diameter and 10.0 mm in length with only 1 open end with the aid of a lentulo spiral. Periapical radiographs were taken to confirm the complete filling of tubes and the absence of bubbles. Eight samples were used for each material. After being filled and weighed, each specimen was immediately immersed in test glass tubes containing 10 mL deionized water, which were then sealed with Parafilm (American National Can, Menasha, WI) and placed in oven at 37°C. The pH was measured with a pH meter (QM-400; Quimis, São Paulo, SP, Brazil) previously calibrated with solutions of known pH (4,7,10). Before the immersion of specimens, the pH of the deionized water was verified to be 6.0. After the removal of the specimens, the test tubes were shaken for 5 seconds before pH measurement. pH evaluations were performed always in fresh tubes containing deionized water at each evaluation period (3, 24, 48, 72, and 168 hours).

-Statistical analysis

Data were statistically analyzed using t-test by means of SPSS software 15.0 (SPSS Inc, Chicago, IL, USA). The signiﬁcance level adopted was *P*<0.05.

## Results

-Cytotoxicity

**Figure 2:**
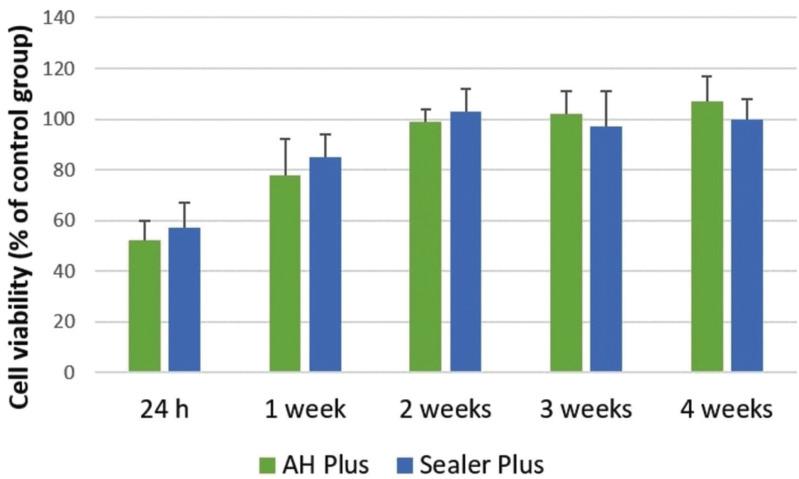
The cytotoxic effects after exposure to sealers in 3T3 fibroblast cells. Results are expressed as mean and standard deviation at different experimental time periods (*P*<0.05).

The results of the MTT assay ovel all the time periods are represented in Figure 2. AH Plus and Sealer Plus showed cytotoxic effects after 24 hours and 1 week of manipulation (*P*>0.05). Both sealers become noncytotoxic after 2 weeks (*P*>0.05).

-Direct contact test

**Figure 3:**
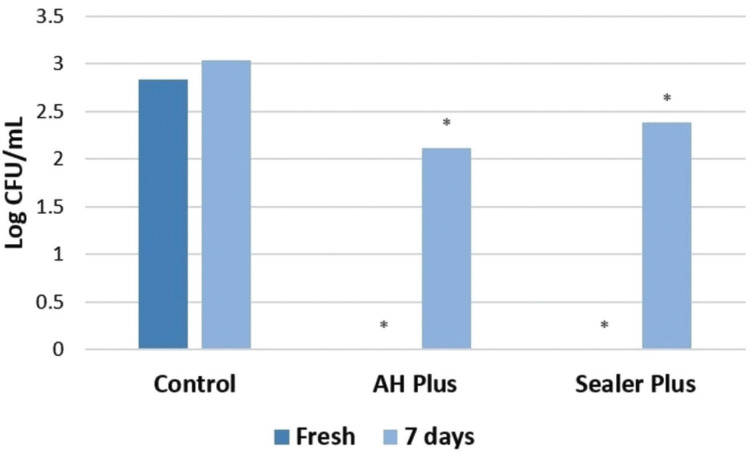
Survival of *Enterococcus faecalis* after direct contact test for 60 min with fresh and aged endodontic sealers. * Value differs significantly from the control group (*P*>0.05) at the same endodontic sealer condition (fresh or aged).

Direct contact test results for the antibacterial effects of the root canal sealers are depicted in Figure 3. Total bacterial eradication was observed for freshly mixed samples of both sealers. However, after 7 days of setting both sealers had lost much of their antibacterial effects.

-Radiopacity

The mean values for radiopacity (mm Al) of the sealers were as follows: AH Plus = 11.64 and Sealer Plus = 7.30. Although statistical analysis showed significant differences between the sealers (*P*<0.05), both sealers showed values complying with the previously mentioned requirements (minimum equivalent to 3 mm Al) (Table 2).

**Table 2:**
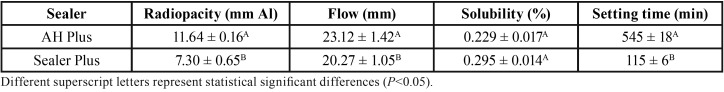
Mean and standard deviations of radiopacity (mm Al), flow (mm), solubility (%) and setting time (min).

-Flow

Although both sealers were in agreement with ISO requirements, AH Plus showed a flow significantly higher when compared to Sealer Plus (*P*<0.05) (Table 2).

-Solubility

AH Plus showed an average weight loss of 0.229%, while Sealer Plus showed a loss of 0.295% (*P*<0.05). Both sealers were in agreement with ISO 6876 statement (Table 2).

-Setting time

AH Plus showed a higher setting time when compared to Sealer Plus (*P*<0.05) (Table 2).

-pH

The pH values at the different evaluation periods are shown in Table 3. AH Plus and Sealer Plus showed a neutral pH at all evaluation periods, with no differences between the sealers (*P*>0.05).

**Table 3:**
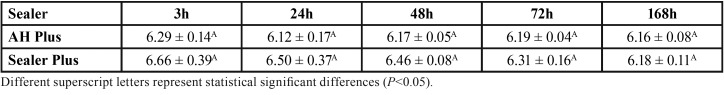
The means and standard deviations of pH values at the different time periods.

## Discussion

The endodontic sealer properties include good sealing ability, adequate radiopacity, easy handling, good resistance, dimensional stability, adequate flow, and low solubility. The present study aimed to evaluate some of the main physicochemical and biological properties of the recently launched endodontic sealer Sealer Plus. It is important to point out that all materials should be tested in a laboratory before being considered for clinical use. However, these tests must attend international standards. International Organization for Standardization (ISO) 6876, 10993-5 and the American Dental Association (ADA) 57 specifications were used in the present study.

A long-term cytotoxicity evaluation was used in the present study. Cytotoxicity testing of freshly mixed sealers is relevant since they are placed into the root canal system in a freshly mixed and incompletely polymerized stage. Nevertheless, it is important to evaluate sealers over extended time periods after setting because, it is probable that during some period after clinical application, changes in cytotoxicity levels may be observed after diffusion of toxic components from the materials into the surrounding environment. According to the present results, both AH Plus and Sealer Plus showed cytotoxicity when cells were exposed to elutes of 24 h and 1 week manipulated sealers. This toxicity decrease over the tested time periods. Our findings are in agreement with previous studies that showed cytotoxicity of AH Plus in the first days after mixing and decreasing over time (7,15), probably as a result of the diminishment in the leaching of toxic substances present in this formulations. A recent study pointed out that Sealer Plus promoted greater cell viability when compared to AH Plus (10); however, differences in the methodologies such as the use different cell lines, time-points and cells exposition to the sealers makes a direct comparison of the studies unfeasible.

The DCT is a quantitative and reproducible method that has been widely used for the evaluation of antimicrobial effects of root canal sealers (14,16,17). Most root canal sealers have weak and short-term antibacterial activity, which significantly decreases after setting (14,17). This was confirmed in the present study for both endodontic sealers. Previous studies also demonstrated that freshly prepared AH Plus demonstrated antibacterial activity, whereas aged samples showed reduced effects against *E. faecalis* (14,16,17). These results are in line with the present results for both endodontic sealers. The toxicity of AH Plus and Sealer Plus against bacteria can be mainly explained by formaldehyde release during setting or the presence of bisphenol A in sealer composition.

Radiopacity is an essential property of endodontic sealing materials. Among other physical, chemical, and biological properties, the ideal root canal sealing material should have a certain degree of radiopacity to be clearly visible on radiographs and enhance the radiopacity of the root filling materials. ISO 6876 standards require a minimal radiopacity equivalent to 3.00 mm Al. In the present study, the radiopacity of both root canal sealers were found to be in agreement with ISO recommendations (12); however, AH Plus showed higher radiopacity when compared to Sealer Plus (*P*<0.05). The tested sealers has one common radiopacyfying agent (zirconium oxide) and two different agents: calcium tungstate in the AH Plus and calcium hydroxide in the Sealer Plus, which might explain the differences among the sealers. Moreover, the differences between radiopacities of the tested sealers could also be explained by different proportions of radiopacifying agents in each root canal sealer, with Sealer Plus having a lower quantity of these agents when compared to AH Plus.

Regarding flow, both sealers showed acceptable values according to ISO recommendations (11) that state the minimal flow required for sealers is 20 mm. Moreover, AH Plus showed significantly superior flow values when compared to Sealer Plus (*P*<0.05). Flow is the ability of a sealer cement to penetrate into the irregularities and accessory canals of the root canal system, and it is considered to be a very important property—the greater the flow, the greater the ability to penetrate into irregularities. Conversely, if the flow is excessive, the risk of material extravasation to the periapex is increased (18).

High solubility of endodontic sealers is undesirable because dissolution may cause release of the materials, allowing formation of gaps between them and the dental structure. Regarding the solubility test, both materials were within the recommended values of ISO standards (11), according to which the tested material should not have solubility greater than 3%. No differences were observed between the tested sealers. AH Plus results are in agreement with previous studies (6,7,15) that showed AH Plus as an endodontic sealer with low solubility. It is important to point out that the solubility testing standards recommend immersion of the materials in water only after complete setting. However, this situation is impossible to be achieved clinically, since the endodontic sealers are immediately placed in the root canal in the presence of moisture. Therefore, solubility values in a clinical scenario are probably higher than the ones found *in vitro*. Recently, a novel method was described to evaluate the dissolution, dislocation and dimensional changes of endodontic sealers using a root canal model and microCT images (19). Using this method, AH Plus showed no significant changes in the percentage volume of material lost and dimensional changes after solubility challenge (19). This method could overcome the limitations of the ISO methodology and could be closed to simulate a clinical scenario.

Endodontic sealers with long setting times are more susceptible to dissolution after root canal filling, whereas extremely short setting times may represent technical difficulties during clinical application. AH Plus showed a higher setting time when compared to Sealer Plus (*P*<0.05). This differences can be explained by differences among resins and the respective hardeners used in each root canal sealer. The AH Plus sealer uses Bisphenol Epoxy Resin, Dibenzyldiamine, Aminodiamantane and Tricychlodecan-diamine, whiles Sealer Plus uses Bisphenol-A Coepichlorohydrin, Bisphenol-F Epoxy Resin and Hexamethylenotetramine.

Alkalinization capacity (increase in pH) may be considered an important chemical property, because it may induce repair by stimulating the mineralization process (20). This question is closely related to the setting time and solubility of the material, as well as the area of the material exposure to the medium in which it is present. Moreover, the chemical characteristics of the material should be considered; that is, whether it has substances that allow the release of hydroxyl ions, and whether it is hydrophobic or hydrophilic. Undoubtedly, a hydrophilic material allows greater contact with the organic fluids to occur. AH Plus and Sealer Plus showed a neutral pH at all evaluation periods, with no differences between the sealers (*P*>0.05). Although Sealer Plus contains calcium hydroxide in its composition, the fast setting time probably prevented the release of hydroxyl ions. The AH Plus does not contain calcium hydroxide in the composition, which justifies the pH values obtained in the present study.

In view of the methodologies used and results obtained, it can be concluded that Sealer Plus presented suitable properties, similar to that obtained by AH Plus sealer.
